# Adult Olfactory Bulb Interneuron Phenotypes Identified by Targeting Embryonic and Postnatal Neural Progenitors

**DOI:** 10.3389/fnins.2016.00194

**Published:** 2016-05-09

**Authors:** Maria Figueres-Oñate, Laura López-Mascaraque

**Affiliations:** Molecular, Cellular, and Developmental Neurobiology, Instituto Cajal, Consejo Superior de Investigaciones CientíficasMadrid, Spain

**Keywords:** *in utero* electroporation, postnatal neurogenesis, postnatal electroporation, periglomerular, lineage tracing

## Abstract

Neurons are generated during embryonic development and in adulthood, although adult neurogenesis is restricted to two main brain regions, the hippocampus and olfactory bulb. The subventricular zone (SVZ) of the lateral ventricles generates neural stem/progenitor cells that continually provide the olfactory bulb (OB) with new granule or periglomerular neurons, cells that arrive from the SVZ via the rostral migratory stream. The continued neurogenesis and the adequate integration of these newly generated interneurons is essential to maintain homeostasis in the olfactory bulb, where the differentiation of these cells into specific neural cell types is strongly influenced by temporal cues. Therefore, identifying the critical features that control the generation of adult OB interneurons at either pre- or post-natal stages is important to understand the dynamic contribution of neural stem cells. Here, we used *in utero* and neonatal SVZ electroporation along with a transposase-mediated stable integration plasmid, in order to track interneurons and glial lineages in the OB. These plasmids are valuable tools to study the development of OB interneurons from embryonic and post-natal SVZ progenitors. Accordingly, we examined the location and identity of the adult progeny of embryonic and post-natally transfected progenitors by examining neurochemical markers in the adult OB. These data reveal the different cell types in the olfactory bulb that are generated in function of age and different electroporation conditions.

## Introduction

In 1962, Joseph Altman revealed that tritiated thymidine could be incorporated into some neurons (based on their appearance under light microscopy) in the rat hippocampus (Altman, 1962; Altman and Das, 1965) and in the olfactory bulb (Altman, 1969). Electron microscopy confirmed the generation of neurons postnatally (Kaplan and Hinds, 1977; Bayer, 1983), a process that involves cell proliferation, neuronal differentiation, and integration into existing neural circuits. Newly generated cells originate in the SVZ of the lateral ventricle, which develops from the residual progenitors of the lateral ganglionic eminence (LGE) at embryonic stages (Bayer et al., 1994; Wichterle et al., 2001; Young et al., 2007). These develop into quite diverse interneurons (Price and Powell, 1970; Pinching and Powell, 1971), characterized morphologically and by their location, firing pattern and immunomarkers. Most of these newly generated interneurons are GABAergic granule cells (GCs: >90%), with a minority differentiating into periglomerular cells (PGCs: Altman, 1969; Luskin, 1993; Lois and Alvarez-Buylla, 1994). To date, PGCs have been characterized either as GABAergic, dopaminergic (Kosaka et al., 1998; Bagley et al., 2007; Batista-Brito et al., 2008), or as a subset of glutamatergic excitatory juxtaglomerular interneurons (Brill et al., 2009; Winpenny et al., 2011). All these PGCs can be incorporated into neural circuits during adulthood, albeit at a lower proportion than granular cells (De Marchis et al., 2007; Whitman and Greer, 2007). The identity of both granule and PGCs is regulated both spatially and temporally (Merkle et al., 2007; Sequerra, 2014; Fuentealba et al., 2015). Moreover, the heterogeneity among newly generated cells is determined in function of their location within the SVZ, defining whether they become granule cells or periglomerular interneurons. Neural progenitor cells (NPCs) in the dorsal adult SVZ give rise to superficial GCs, CR^+^ cells, and periglomerular TH^+^ cells, whereas the lateral and ventral regions generate mostly deep GCs and periglomerular Calbindin^+^ cells (for a review, see Fiorelli et al., 2015). In addition, the diversity of olfactory bulb (OB) interneurons is also determined by their temporal origin (De Marchis et al., 2007). TH+ and Calbindin+ (PGCs) production are generated principally during embryogenesis and their production declines postnatally, when Calretinin^+^ GCs and PGC generation increases (Batista-Brito et al., 2008). Specific subpopulations of adult newborn OB cells have also been characterized based on progenitor domains (Merkle et al., 2007, 2014). Adult born GCs have been classified into five different groups based on their maturational states (Petreanu and Alvarez-Buylla, 2002) and new subtypes are still being described (Merkle et al., 2014).

In order to fully capture the heterogeneity among the OB interneurons generated in the adult, embryonic, or postnatal cells were targeted by electroporation with a ubiquitous transposable vector expressing the enhanced green fluorescent protein (eGFP). Our data reveals this tool to be a powerful means to visualize the specific cell fate of different embryonic and postnatal progenitors in function of their age and on the placement of the electrodes for electroporation. Thus, in this analysis we are able to correlate, lineages, and cell dispersion within the different OB layers as a function of those parameters.

## Materials and methods

### Animals

Wild type C57BL/6 mice were obtained from the Cajal Institute animal facility, and the day the vaginal plug was detected was considered as the first embryonic day (E0) and day of birth as postnatal day 0 (P0). All procedures were carried out in compliance with the ethical regulations on the use and welfare of experimental animals of the European Union (2010/63/EU) and the Spanish Ministry of Agriculture (RD 1201/2005 and L 32/2007), and the CSIC's bioethical committee approved all the animal protocols. At least *N* = 3 animals were used per experimental condition.

### Transposable vectors

The pPB-UbC-eGFP integrable plasmid containing eGFP under the control of the human ubiquitous Ubiquitin C (UbC) promoter was used here (Yusa et al., 2009; Figueres-Oñate et al., 2015). The genomic integration of this construct was mediated by co-electroporation with a vector containing a hyperactive transposase of the PiggyBac system (hyPBase), kindly provided by Prof. Bradley (Yusa et al., 2011).

### *In utero electroporation* (IUE)

Briefly, E13 or E15 pregnant mice were anesthetized with 2% isofluorane (Isova vet, Centauro), which was maintained with 1.5% isofluorane/O_2_ inhalation, and the mice were placed on a thermal plate at 37°C during the surgery. Before making the peritoneal excision, the mice were administered an antibiotic, enrofloxacin (5 mg/kg: Baytril Bayer), and an anti-inflammatory agent, meloxicam (300 μg/kg: VITA Laboratories). The uterine horns were exposed by midline laparotomy and the embryos maintained humid with physiological saline. Embryos were visualized by trans-illumination and up to 1 μl of the plasmid mixture was injected through a pulled glass capillary into the lateral ventricle of each embryo. The DNA mixture was comprised of the pPB-UbC-eGFP construct and hyPBase in a 3:1 ratio (1–2 μg/ml final concentration), with 0.1% Fast Green (SIGMA). Five consecutive electric square wave pulses (33 or 37 V, 50 ms duration, 950 ms interval) were then applied with 3 mm tweezer-type electrodes (Sonidel). Subsequently, the uterine horns were returned to the abdomen, and the abdominal muscle and skin were sutured. The mice were allowed to recover at 37°C with oxygen administration.

### Postnatal electroporation

Pups (P0/P1) were anesthetized by hypothermia and placed under a cold light to facilitate visualization of the lateral ventricles by trans-illumination. The DNA mixture (see above) was injected into the ventricular cavity and electroconductive LEM Gel (DRV1800, MORETTI S.P.A.) was placed on both electrode paddles to avoid damaging the pups and to achieve successful current flow. Five 100 V electric pulses were applied (50 ms duration, 950 ms intervals), with the positive electrode positioned in the dorso-lateral region to direct the negatively charged DNA to the subventricular zone. After the pulses, the pups were placed on a thermal plate and when they had recovered, they were returned to their mother.

### Tissue processing

Brains were analyzed at adult stages (from P25 onwards). Mice were deeply anesthetized by intraperitoneal injection of sodium pentobarbital (Dolethal, 40–50 mg/kg) and they were transcardially perfused with 4% paraformaldehyde (PFA). The brains were post-fixed in PFA overnight at 4°C and 50 μm thick free-floating coronal or sagittal vibratome sections were obtained.

### Immunohistochemistry

To identify the phenotypes of the olfactory bulb cell population, we studied the immunohistochemical labeling of neuronal and glial antibodies. Sections were permeabilized with 0.1% Triton-X in PBS (PBS-T), blocked with 5% normal goat serum (NGS) and then they were incubated with the primary antibodies (see Table 1). The following day, the sections were washed and subsequently incubated with the appropriate conjugated secondary antibodies: red fluorophore (1:1000, Alexa Fluor 568 nm, Molecular Probes) or an infrared fluorochrome (1:1000, Alexa Fluor 633 or 647 Molecular Probes).

**Table 1:** **Primary antibodies used for the immunohistochemical analysis of newly generated cells in the adult olfactory bulb**.

**Antibody**	**Abbreviations**	**USE**	**SP**.	**Source**
Adenomatous Polyposis Coli	APC/CC-1	1:200	MS	Calbiochem (OP80)
Calbindin	CB	1:500	Rb	Abcam (Ab11426)
Calretinin	CR	1:500	Rb	Abcam (Ab702)
2′,3′-Cyclic-nucleotide 3′phosphodiesterase	CNPase	1:200	Ms	Covance (SMI-91R)
Doublecortin	DCX	1:500	Rb	Cell signaling (4604)
DOPA decarboxylase	DDC	1:500	Rb	Abcam (Ab3905)
Gamma-Aminobutyric acid	GABA	1:500	Rb	Sigma (A2052)
Glutamate decarboxylase 67	GAD67	1:500	Ms	Millipore (MAB5406)
Glial fibrillary acidic protein	GFAP	1:1000	Ms	Millipore (MAB3402)
Myelin Basic Protein	MBP	1:500	Rat	Serotec (MCA409S)
Neuronal Nuclei	NeuN	1:500	Ms	Millipore (MAB377)
Neuron-glial antigen 2	NG2	1:500	Ms	Millipore (AB5320)
Oligodentrocyte transcription factor 2	Olig2	1:2000	Rb	Millipore (AB9610)
Alpha-type platelet-derived growth factor receptor	PDGFRα	1:300	Rb	Santa Cruz (C-20) (sc-338)
Parvalbumin	PV	1:500	Rb	Swant (PV-25)
Reelin	Reel	1:500	Ms	Millipore (MAB5364)
S100 calcium binding protein beta	S100β	1:300	Ms	Abcam (Ab66028)
Somatostatin	SOM/SST	1:500	Rb	Millipore (AB5494)
T-box, brain, 1	Tbr1	1:500	Rb	Abcam (AB31940)
T-box, brain, 2	Tbr2	1:500	Rb	Abcam (Ab23345)
Tyrosine hydroxylase	TH	1:500	Rb	Millipore (AB152)

### Imaging processing

Green fluorescent labeling was examined under an epifluorescence microscope (Nikon, Eclipse E600) with the fluorescein filter cube (450–490 nm, Semrock). Immunohistochemical labeling was observed with the red and far-red filter cubes: rhodamine (569–610 nm) and Cy5 (628–640 nm). The final images were acquired on a Leica TCS-SP5 confocal microscope adjusting the settings so that there was no spectral overlap: eGFP (Ex: 488; Em: 498–550), Alexa 568 (Ex: 561; Em: 575–620), and Alexa 633/647 (Ex: 633; Em: 645–740). Confocal laser lines were used in-between 25 and 40% in all cases.

The maximum projection images were created using the confocal software (LASAF Leica) and other software, such as NIH-ImageJ software. Captured images were processed to adjust the contrast and brightness equally using Adobe Photoshop CS5 software.

### Quantitative analysis

After image acquisition the distribution of the OB cells after either postnatal or *in utero* electroporation was analyzed with ImageJ software. First, images were converted to 8-bits and to facilitate the analyses of both the cell's morphology and layer localization, ICA Lut was applied. Cells were counted using the Cell Counter plug-in analysis tool of the Image J software, analyzing four P25-30 mice animals per group (IUE or postnatal electroporation). Up to 1875 OB cells were counted following postnatal electroporation and 926 cells from the *in utero* electroporated animals. The somata area of DCX positive cells was analyzed with the ImageJ software (60 cells within the granular cell layer and 40 within the subependymal zone). Statistical analyses were performed with SigmaPlot 13 (Systat Software) and the values were represented as the mean ± SEM. *T*-tests were performed to determine the significance between different groups, or with the Mann-Whitney Rank Sum Test when the normality test failed. A confidence interval of 95% (*p* < 0.05) was required to considered values statistically significant.

## Results

### Histochemical phenotypes of neural olfactory bulb cells

To establish the profile of neurochemical markers expressed by OB interneurons, first we performed an immunohistochemical study for a battery of markers (Table 1 and Supplementary Figures S1, S2). Both neuronal and glial markers were used to determine the heterogeneity of cell phenotypes from the glomerular layer (GL), external plexiform layer (EPL), mitral layer (ML), internal plexiform layer (IPL), granular cell layer (GCL) to the subependymal zone (SEZ; Supplementary Figure S1A). Calbindin (CB, Supplementary Figure S1B) immunoreactive cells appeared predominantly in both the GL and the external plexiform layer (EPL). However, tyrosine hydroxylase (TH) was exclusively expressed by a subset of PGCs (Supplementary Figure S1C). Calretinin (CR, Supplementary Figure S1D) immunolabeling was restricted to periglomerular and granular cells, while Parvalbumin^+^ cells (PV, Supplementary Figure S1E) were located throughout the EPL and occasionally in the external granular cell layer (eGCL). Few somatostatin (SOM) interneurons were found in the EPL (Supplementary Figure S1F), while dopa decarboxylase (DDC) was expressed by a large number of periglomerular and granular cells (Supplementary Figure S1G). All the interneurons were generally labeled for either GABA (Supplementary Figure S1H) or GAD67 (Supplementary Figure S1I). The microtubule-associated protein doublecortin (DCX), that plays an important role in neuronal migration, labeled neuroblasts within the SEZ and throughout the GCL (Supplementary Figure S1J). The extracellular protein reelin was mainly detected around mitral cells but also, around some tufted and periglomerular cells (Supplementary Figure S1K). Interestingly, the regulatory T-box brain 1 and 2 genes (Tbr1 and Tbr2) were expressed by a few glutamatergic periglomerular OB cells. Indeed, while both were expressed at the same OB location, Tbr2 labeled a few more cells (Supplementary Figure S1L) than Tbr1 (Supplementary Figure S1M) as described previously (Winpenny et al., 2011). To tag all the neurons in the OB we used the marker of neuronal nuclei, NeuN, which was mostly expressed in the GCL and the mitral cell layer in the OB (Supplementary Figure S1N). As a large number of neurons, mostly in the GL, did not express NeuN, we assessed the neurochemical identity of NeuN by double staining with CB (Supplementary Figure S1O) and CR (Supplementary Figure S1P). Although most CB^+^ cells in the GL did not express NeuN, both antibodies co-stained a population of GL neurons, (arrowheads in Supplementary Figure S1O magnification). Only a few cells were dual stained for CR and NeuN in the GCL (arrowheads in Supplementary Figure S1P magnification). To further address the phenotype of the cells expressing NeuN, dual NeuN, and DCX labeling was assessed, the later an early neural marker (Supplementary Figure S1Q). While most DCX cells concentrated in the SEZ, both markers co-localized in a subset of GCL cells close to the SEZ (arrowheads in Supplementary Figure S1Q magnification), indicating an overlap of those immunomarkers. Finally, to better understand the distribution of the mature interneurons and the neuroblasts migrating along the SVZ-RMS-OB pathway, double immunostaining with CR-DCX was performed. DCX positive cells were confined to the RMS and the SEZ, while CR^+^ cells were located in the eGCL and GL, regions with weaker DCX labeling (Supplementary Figure S1R).

The distribution of glial cells in the OB was studied with markers for astrocytes (GFAP and S100β proteins), NG2 cells (antibodies against NG2 and PDGFRα), and oligodendrocytes (Olig2, MBP, APC, and CNPase). GFAP-positive cells were located throughout the OB layers except for the EPL, mostly concentrating in the SEZ (Supplementary Figure S2A). NG2 stained cells were homogeneously distributed throughout all the OB layers (Supplementary Figure S2B) and Olig2 immunoreactivity was equally widespread along the SVZ-RMS-OB pathway (Supplementary Figure S2C). Finally, CNPase expression was restricted to myelinating oligodendrocytes (Supplementary Figure S2D). Dual immunofluorescence for Olig2 and APC (Supplementary Figure S2E) indicated that the vast majority of APC^+^ cells co-expressed Olig2 (arrowheads in Supplementary Figure S2E magnifications), although since Olig2 is a marker for the complete oligodendroglial lineage and APC only labels myelinating oligodendrocytes, not all cells with Olig2 expressed the APC marker (asterisks in Supplementary Figure S2E magnifications). Olig2 and MBP did not co-localize (Supplementary Figure S2F) as their expression in oligodendrocytes differs considerably: Olig2 labels the nucleus while MBP is found in myelin sheaths. Finally, we also analyzed the co-expression of oligodendroglial and astroglial lineages with the combinations of the S100β-Olig2 (Supplementary Figure S2G) and GFAP-PDGFRα (Supplementary Figure S2H) markers. S100β and Olig2 were expressed in different cell populations (asterisks in Supplementary Figure S2G magnifications), although the S100β astrocytic marker co-localized with some Olig2^+^ oligodendrocytes (arrowheads in Supplementary Figure S2E magnifications). By contrast, the GFAP and PDGFRα immunomarkers (Supplementary Figure S2H) did not co-localize along the SVZ-RMS-OB pathway (Supplementary Figure S2Ha magnification). In fact, GFAP was expressed strongly within the RMS (Supplementary Figure S2Hb magnification) while PDGFRα maintained a uniform cell density through this migratory channel.

### Tracking different olfactory bulb cell lineages with different electroporation conditions

**Figure 1:**
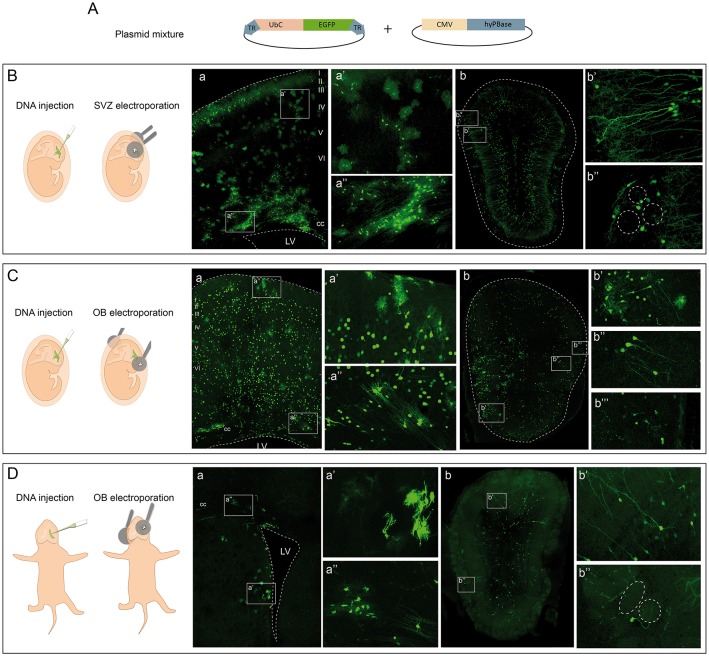
**Targeting cells in the olfactory bulb by electroporation. (A)** *In vivo* electroporation after ventricular injection of a plasmid mixture containing an integrable vector expressing the eGFP reporter and the transposase of the PiggyBac system. **(B)** Dorso-lateral *in utero* electroporation (IUE) after injection of the plasmid mixture into the lateral ventricles. **(a)** Transfected cells occupying several layers of the cerebral cortex and the corpus callosum (cc) in adult mice. In particular, glial cells with different morphologies are shown, astrocytes in **(a**′**)** and oligodendrocytes in **(a**″**)**. After IUE of the subventricular zone (SVZ) OB cells were found in the OB **(b)** corresponding to granular **(b**′**)** and periglomerular **(b**″**)** phenotypes. **(C)** Rostral IUE after injection of the plasmid mixture into the lateral ventricles labeled cells in the cerebral cortex **(a)**. Neurons were widespread throughout the cerebral cortex, as were astrocytes **(a**′**)**. Cells corresponding to oligodendrocytes were positioned in the cc **(a**″**)**. Cells in the OB **(b)** with a glial **(b**′**)** and neuronal morphology corresponding to granular **(b**″**)** and periglomerular cells **(b**^‴^**)**. **(D)** Postnatal (P1) electroporation of dorso-lateral SVZ-progenitors. Labeled cells were surrounding the ventricular surface **(a)** with astroglial **(a**′**)** and oligodendroglial **(a**″**)** morphologies. In the OB **(b)** cells with a neuronal phenotype were situated in the granular **(b**′**)** and glomerular **(b**″**)** layers.

We followed the cell progeny in the OB by targeting neural progenitors using *in utero* or postnatal electroporation of the following vectors: a plasmid ubiquitously expressing eGFP flanked by two terminal repeat sequences (TRs); the PiggyBac transposase (mPBase). The mPBase transposase integrates the vector including the reporter gene directly into the genome of the transfected cell (Figure 1A), allowing the entire progeny of a single cell to be analyzed, regardless of its mitotic activity. To label the different OB populations, IUE was performed (E13–E15) varying electrode positions and with distinct orientations along rostro-caudal axis. Interestingly, neural populations labeled were located at different positions in the OB depending on the embryonic area electroporated. First, IUE was performed at E15 to target progenitors from the dorso-lateral area of the ventricular surface (Figure 1B). Analyzing brains 30 days post-electroporation (dpe), labeled cells corresponding to glial and neuronal lineages were located within the dorsal cortex (Figure 1Ba). After electroporation on E15, neurons were located within cortical layers II-III, whilst glial cells were widespread throughout cerebral cortex (Figure 1Ba′). Moreover, neuronal precursors and labeled oligodendrocytes were located within the corpus callosum, near to the ventricular surface (Figure 1Ba″). Furthermore, a large population of labeled neurons was also evident in the OB (Figure 1Bb). Transfected neurons were widespread along the rostro-caudal and ventro-lateral OB axis at 30 dpe, mostly located within the GCL (Figure 1Bb′) and GL (Figure 1Bb″). Remarkably, no glial cells were detected in the OB when performing dorso-lateral electroporations.

To assess the potentiality of progenitors at the OB ventricular surface, E13 IUE was performed directing the electrodes to the most rostral part of the LV (Figure 1C). At adult stages (30 dpe), labeled cells were distributed within the cerebral cortex of E13 electroporated brains (Figure 1Ca), with neurons distributed within layers II-VI of the cerebral cortex and glial cells also occupying several cortical areas (Figure 1Ca′). Progenitors remained labeled in the SVZ, as well as recognizable oligodendroglial cells (Figure 1Ca″). In contrast to dorso-lateral electroporation, labeled glia, and neurons were evident in the OB (Figure 1Cb). Thus, OB glial cells were produced by progenitors from the rostral part of the ventricular surface (Figure 1Cb′) but not by dorso-lateral progenitors after *in utero* electroporation (Figure 1Bb). The identity of the OB glia was addressed by studying different markers, corroborating the presence of astrocytes through the co-localization of eGFP with GFAP (Supplementary Figure S3A) and S100β (Supplementary Figure S3B). The oligodendroglial lineage was identified by either Olig2 (Supplementary Figure S3C) or PDGFRα (Supplementary Figure S3D), and electroporated cells that co-expressed both were present in the adult OB after such electroporations. Moreover, granular (Figure 1Cb″) and periglomerular cells (Figure 1Cb^‴^) were also labeled in the GCL and GL of the adult OB. Regarding their distribution, it is important to note that glial cells were located only within a lateral region of the OB, while neurons were broadly dispersed across the whole OB. Thus, neurons and glia generated from embryonic progenitors appear to have a different distribution pattern. Labeled SVZ progenitors remained at the adult ventricular surface (Figure 1Ca″), suggesting that some interneurons may still originate from these SVZ-transfected progenitors in the adult brain.

Finally, to specifically analyze the postnatal contribution of SVZ progenitors to the OB, we performed postnatal (P1) electroporations positioning the electrodes in the dorso-lateral region (Figure 1D). Transfected progenitors were evident in the dorso-lateral area of the ventricular surface of adult brains (30 dpe: Figure 1Da). Moreover, glial cells surrounded the electroporated area near the ventricular surface, with astrocytes (Figure 1Da′) and oligodendrocytes (Figure 1Da″) readily recognized. Targeting postnatal progenitors in the dorso-lateral region labeled interneurons in the OB (Figure 1Db). These postnatally labeled interneurons were mostly GCs located in the GCL (Figure 1Db′) and PGCs surrounding the glomeruli (Figure 1Db″).

### Cell phenotypes after embryonic and postnatal electroporation

**Figure 2:**
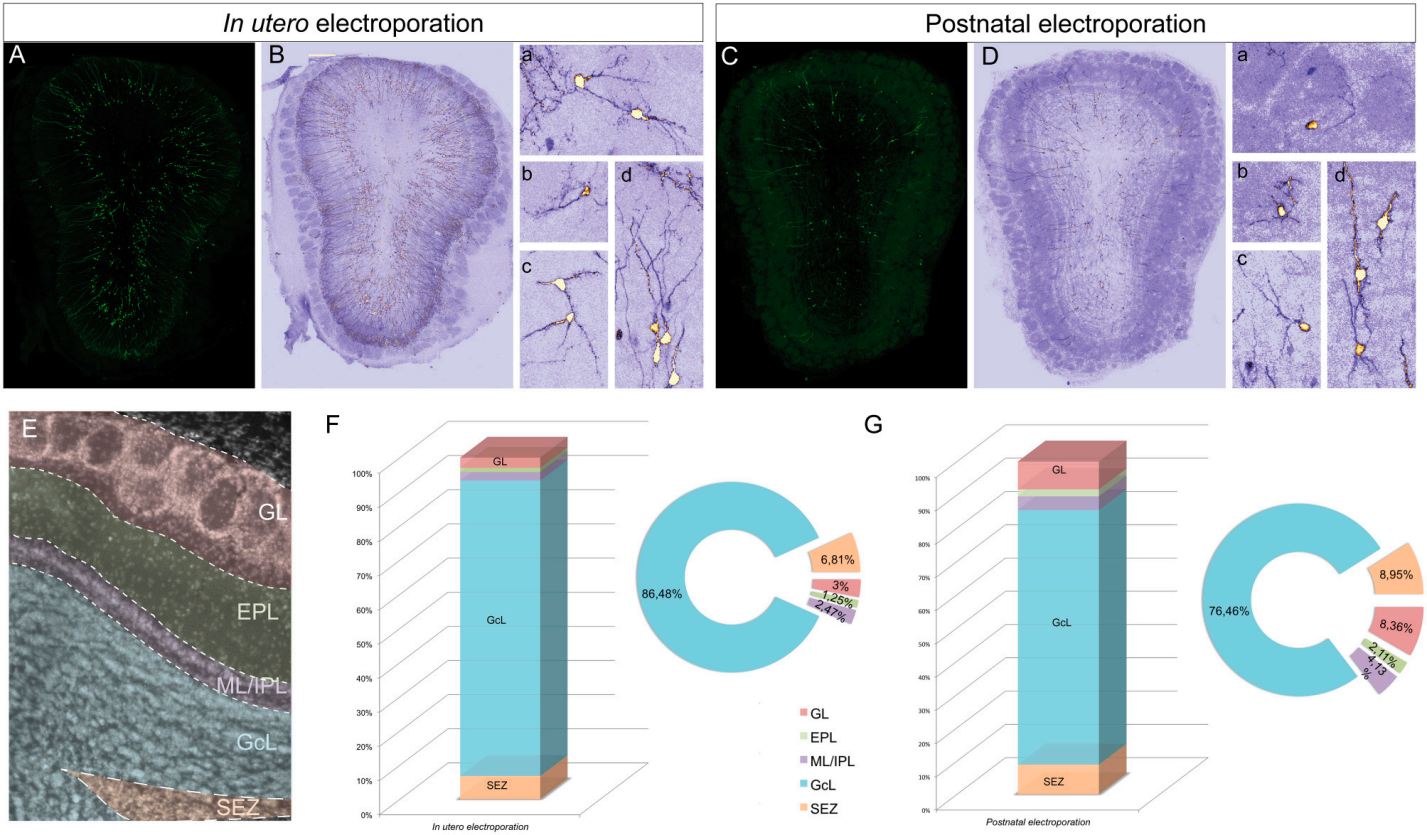
**Distribution of electroporated cells within the OB**. The percentage of electroporated cells occupying different layers of the OB was studied after electroporation at E15 IUE or P1. **(A)** Representative coronal section of a P30 animal electroporated at E15. **(B)** To quantify the distribution the images were analyzed with ICA Lut (ImageJ software). Representative morphologies of periglomerular **(Ba)** and granular **(Bb–d)** electroporated cells. **(C)** Representative slice of a P30 OB after P1 electroporation. **(D)** Different cell morphologies and dispersion analysis was performed by applying the ICA Lut. Particular morphologies of periglomerular **(Da,b)** and granular **(Db–d)** electroporated cells are showed. **(E)** Different layers of analyzed OB: GL, glomerular layer; EPL, external plexiform layer; ML/IPL, mitral and internal plexiform layer; GCL, granular cell layer; SEZ, subependymal zone. **(F,G)** The percentage of P30 labeled cells after E15 IUE (1875 cells in 4 animals) and P1 electroporation (926 cells in 4 different animals) are shown in **(F,G)**, respectively.

To assess the distribution and heterogeneity among the eGFP-transfected cells after *in utero* or postnatal electroporation, the cell dispersion within the OB layers was quantified at P30 (Figure 2). To focus exclusively on the extrabulbar origin of OB interneurons, only dorso-lateral electroporations of the SVZ were studied, examining a total of four animals with similar electroporations for both analyses (Figures 2A,C). A gross morphological analysis with ICA Lut (ImageJ) distinguished different arbor morphologies (Figures 2B,D) mainly associated with the glomerular (Figures 2Ba,b,2Da,b) and granule cell (Figures 2Bc,d,2Dc,d) layers. Glomerular cells have oval or round-shaped cell bodies, giving rise to a spiny apical dendritic tree that arborizes within the glomerulus (Figures 2Ba,b,2Da,b). However, most cells were overwhelmingly restricted to the GCL and they had dendritic arbors with a characteristic GC morphology (Figures 2Bc,d,2Dc,d). We also quantified the percentage of electroporated cells in each layer of the OB after *in utero* or neonatal electroporation. Cells were arranged into five groups regarding their position within the GL, EPL, ML/IPL, GCL, or SEZ (Figure 2E). In general, eGFP-positive cells were similarly distributed after *in utero* or postnatal electroporation (Figures 2F,G). The proportion of the adult labeled cells in the different OB layers was quantified following *in utero* (*n* = 4 animals, 1875 eGFP-adult cells) and P1 (*n* = 4 animals, 926 eGFP-adult cells) electroporation. The vast majority of cells were located within the GCL (IUE 86.48 ± 0.59 and 76.46 ± 3.2% postnatal electroporation), with E15 and P1 electroporated animals displaying fewer labeled cells in the EPL (IUE: 1.25 ± 0.18% and P1: 2.11 ± 0.31%) and ML/IPL (IUE: 2.47 ± 0.32% and P1: 4.13 ± 0.96%). Thus, no significant differences were found in the layer distribution of the labeled cells between IUE and postnatal electroporation. However, the percentage of labeled cells in the GL was significantly different between E15 and P1 electroporated animals (*t*-test, *P* = 0.022), with more labeled cells in the GL of postnatal electroporated animals (8.36 ± 1.65%) than in electroporated embryos (3 ± 0.22%).

**Figure 3:**
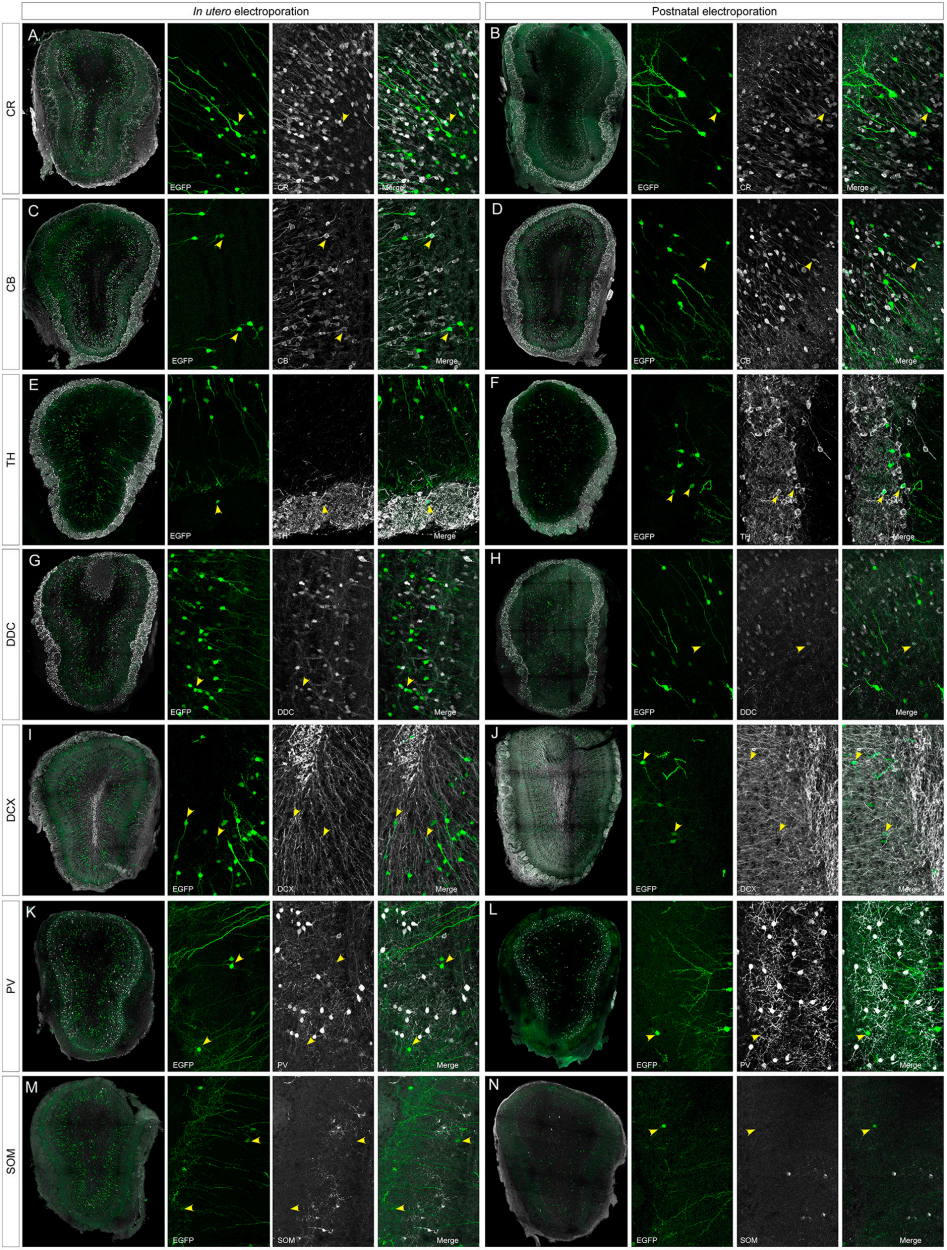
**Neuronal immunolabeling after IUE (E15) or postnatal (P1) electroporation**. At P30, EGFP labeled cells immunostained for: CR, calretinin **(A,B)**; CB, calbindin **(C,D)**; TH, tyrosine hydroxylase **(E,F)**; DDC, dopa decarboxylase **(G,H)**; DCX, doublecortin **(I,J)**. Arrowheads in magnifications show targeted cells that co-express the respective markers. **(K–N)** After IUE or postnatal electroporation, analyzed cells targeted in the OB (arrowheads in **K,L**) did not contain parvalbumin (PV, arrowheads in **K,L** magnifications) or somatostatin (SOM, arrowheads in **M,N** magnifications).

**Figure 4:**
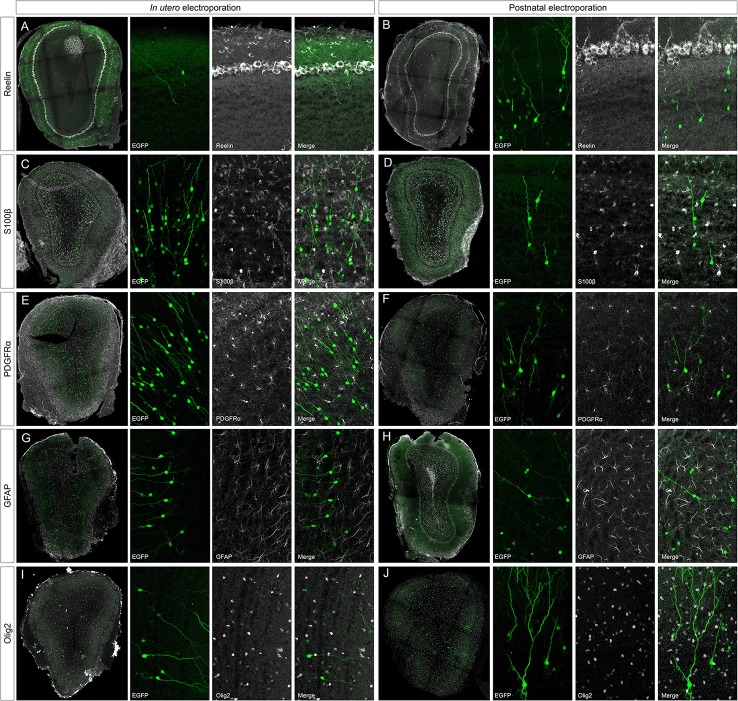
**Negative immunomarkers after IUE or postnatal electroporations. (A,B)** After E15 or P1 electroporation, adult targeted cells (P30) did not express reelin (reel). **(C–J)** The analyzed glial markers, S100β **(C,D)**, PDGFRα **(E,F)**, GFAP **(G,H)**, or Olig2 **(I,J)**, did not co-localized with P30 EGFP^+^ cells. Thus, neither projection nor glial cells were targeted after electroporation of the dorso-lateral progenitors from the ventricular zone at embryonic or postnatal stages.

To address the heterogeneity of the cells generated by targeted progenitors we analyzed the neurochemical markers expressed by labeled cells within the OB (Figure 3). As our specific aim was to analyze interneurons that contribute to the olfactory bulb from an extra bulbar origin throughout life, electroporation was performed to target the dorso-lateral SVZ in either embryos (Figure 1B) or postnatal (Figure 1D) mice. The eGFP expressing cells analyzed in P30 mice represented a heterogeneous population of interneurons that expressed CR (Figures 3A,B), CB (Figures 3C,D), TH (Figures 3E,F), DDC (Figures 3G,H) and DCX (Figures 3I,J), yet not reelin (Figures 4A,B) or glial markers (Figures 4C–J).

According to the literature, the dorso-lateral area targeted is the origin of TH positive PGCs and thus, after electroporation, labeled cells were distributed in the glomerular area of the OB (Figures 2F,G). The number of EGFP^+^ periglomerular cells was significantly higher in postnatal electroporated animals. However, the vast majority of eGFP-labeled cells were located within the GCL and SEZ as electroporation was directed toward the most dorso-lateral ventricular zones. In the OB, the labeled cells corresponded to a highly heterogeneous population, as reflected by the co-expression of the selected neuronal markers (arrowheads in Figures 2A–H magnifications), although interestingly in low numbers. The distribution of the labeled cells did not significantly vary after embryonic or postnatal electroporation, excluding PGCs. Regarding to the maturation of targeted cells, eGFP-DCX cells were found within the SEZ layer after either embryonic or postnatal electroporation (Figures 3I,J arrowheads). There were significant differences in the soma size of DCX positive cells in the SEZ (white region in Figure S4A) and GCL (pink area in Figure S4A), the former representing significantly smaller cells within the SEZ (mean 23.02 ± 0.89 μm^2^, Supplementary Figure S4B) than those in the GCL (mean 51.97 ± 1.12 μm^2^, Supplementary Figure S4C: Mann–Whitney test, *P* < 0.001, Supplementary Figure S4D). This suggests some maturation of the SEZ output when it reaches its final destination. In addition, after E15 IUE eGFP-DCX positive cells at P30 were found either near the SEZ, in the inner GCL, in the outer GCL or in the GL, reflecting the wide distribution of immature cells at the stages analyzed (Supplementary Figures S4E–G).

After IUE and postnatal electroporation transfected cells were rarely located in the EPL (IUE 1.25 ± 0.18 and 2.11 ± 0.31% postnatal electroporation) where PV and SOM are expressed widely. Moreover, the few eGFP-labeled cells in this layer did not express either PV (arrowheads in Figures 3K,L magnification) or SOM (arrowheads in Figures 3M,N magnification). We also performed reelin immunostaining to verify that no projection neurons were targeted (Figures 4A,B). Since mitral cells are generated between E10 and E13, with a peak of genesis at E11 (Blanchart et al., 2006), no eGFP-positive mitral cells were detected when IUE was performed at E15, as shown by reelin expression (Figures 4A,B). As expected, eGFP did not co-localize with any of the glial markers analyzed (S100β, Figures 4C,D; PDGFRα, Figures 4E,F; GFAP, Figures 4G,H; Olig2, Figures 4I,J) as electroporation did not target progenitors within the olfactory ventricle (Figure 1C).

## Discussion

This study addressed the distribution and neurochemical identity of adult OB interneurons targeted at either embryonic or postnatal ages with a ubiquitously expressed transposable reporter vector encoding eGFP. Through this approach, a stable tag is introduced into the genome of targeted progenitor cells that is inherited by all their progeny (Figueres-Oñate et al., 2015). Our results showed that the age of the mice and the electrode placement was critical for the targeting of different cell lineages in the OB, particularly for glial lineages and projection neurons. Moreover, the targeted cells represented a heterogeneous population of interneurons, both in animals electroporated as embryos or postnatally. The distribution of the progeny of either embryonic or postnatal dorso-lateral progenitors was similar in all the OB layers, except in the glomerular area where more PGCs were labeled by postnatal electroporation.

### A comparison of embryonic and postnatal OB interneuron generation

We previously reported that after *in vivo* embryonic electroporation of SVZ progenitors, several labeled OB interneurons undergo rounds of cell divisions before they differentiate (Figueres-Oñate et al., 2015). The progenitors of these interneurons were present in the SVZ at embryonic stages and the embryonic time at which the different OB populations of projection neurons and interneurons are generated has been addressed through *in utero* electroporation (Chen and LoTurco, 2012; Imamura and Greer, 2013, 2014; Siddiqi et al., 2014). OB interneurons originate from E13 progenitors located in the sub-pallium, specifically in the LGE (Wichterle et al., 2001; Kohwi et al., 2007). These LGE progenitors later distribute along the ventricular surface and some of them become adult neural stem cells, this destiny being specified before E15 (Fuentealba et al., 2015). Accordingly, our dorso-lateral electroporation at E15 labeled SVZ progenitors that were already compromised to establish certain OB interneuron subtypes. The temporal pattern of interneuron generation from embryonic to postnatal stages has been studied by tracing the lineages derived from specific progenitors in transgenic mice (Calzolari et al., 2015). In this way it became clear that different OB interneuron populations are generated from DLX1/2 progenitors at different times (Batista-Brito et al., 2008). Different adult new-born subpopulations are generated at different ages, the most heterogeneous being generated around the time of the animal's birth, when olfactory sensation begins (Brann and Firestein, 2014). For example, CB-positive PGCs are preferentially generated during the early postnatal period, whereas CR and TH neurons are mainly produced later in life (De Marchis et al., 2007). To measure the potential differences between OB interneurons generated at postnatal or embryonic ages, we used *in utero* and postnatal electroporation. However, at P30 there were no significant differences between the populations of transfected cells after dorso-lateral embryonic or postnatal electroporation. The postnatal generation of specific granular or periglomerular interneuron subpopulations that originate from specific Pax6, Tbr2, 5HT3, or Neurog2 progenitors has previously been studied in the OB (Kohwi et al., 2005; Inta et al., 2008; Brill et al., 2009; Winpenny et al., 2011). Two weeks after postnatal electroporation at the ventricular surface, OB cells that were labeled expressed markers of interneurons and they were electrically excitable (Chesler et al., 2008). Moreover, the heterogeneity and specific regionalization of these interneurons in the OB can be addressed changing the orientation of the electrodes (Fernández et al., 2011). Thus, the SVZ represents a heterogeneous pool of neural progenitors at both embryonic or postnatal stages, highlighting that a correlation exists between OB neuron-type and SVZ regionalization (Hack et al., 2005; Merkle et al., 2007).

With regards location, our data showed that there were few transfected cells within the EPL and ML/IPL, the vast majority occupying either the most external or internal part of the granular cell layer. The increase in the proportion of transfected PGCs after postnatal electroporation may be related to the precise location of the SVZ progenitors that differentially contribute to distinct types of periglomerular interneurons (Lledo et al., 2008).

### Targeted cells do not co-localize with a large number of markers

Our data show that newborn cells represent a heterogeneous population, since the transfected eGFP-cells expressed most of the markers analyzed. Actually, the large variety of markers for newly generated cells contrasts with the lower number of newborn cells expressing each when compared with the total number of eGFP transfected cells. In fact, after virus infection (Merkle et al., 2007), *in utero* electroporation (Fernández et al., 2011), or tamoxifen administration in transgenic mice (Batista-Brito et al., 2008), the percentages of labeled GCs that express immunohistochemical markers is also really low. Those different approaches to label adult newborn cells in the OB established that the percentage of CR and CB expressing neurons in the GCL is no more than 20% (Bagley et al., 2007). However, the percentage of positive CR cells in relation to the total population of cells in the GCL of the adult OB is around 10% (Parrish-Aungst et al., 2007). Therefore, despite the large diversity of markers used there are relatively few newly generated cells with regards the total proportion of those cells types in the OB. In this respect, and due to the lack of markers, new cells occupying the internal part of the granular layer were described by their morphology, layering, and origin (Merkle et al., 2014). This highlights the need for new markers to define these populations of newborn interneurons that are generated in the OB throughout adulthood.

Another possibility is that those cells remained immature at the time of analysis (P30), although newly generated cells display electrical properties 2 weeks after postnatal electroporation (Chesler et al., 2008). Similarly, Tbr1-Tbr2 expression is evident in dopaminergic cells 21 days after labeling (Winpenny et al., 2011). In other studies the immunochemical nature of OB interneurons arising from ventricular-targeted progenitors can be defined 15–30 days post progenitor targeting (Fernández et al., 2011; de Chevigny et al., 2012; Merkle et al., 2014). Otherwise, we found labeled cells that expressed immature markers like DCX at 20 dpe, widely spread across the OB, although there were too few to assume that analyzed cells did not express mature markers because they were undifferentiated. Moreover, our dual immunohistochemistry studies using markers of mature (NeuN, CB, CR) and immature (DCX) cells showed some overlap within these populations, indicating a gradual maturation of these cells.

### Ontogeny of glial cells in the olfactory bulb

We recently analyzed the clonal dispersion and migratory routes of NG2 cells in the OB (García-Marqués et al., 2014) using the StarTrack method (García-Marqués and López-Mascaraque, 2013). We also described the large heterogeneity within the glial lineages in the OB (Figueres-Oñate et al., 2014; García-Marqués and López-Mascaraque, 2016) and cerebral cortex (Bribián et al., 2016). However, the origin of glial cells in the OB is not as well understood as that of neurons. Most studies describe the glial populations in the OB through their morphology (De Castro, 1920; Valverde and Lopez-Mascaraque, 1991) or using immunohistochemical markers (Bailey and Shipley, 1993; Chiu and Greer, 1996; Emsley and MacKlis, 2006), while the origin and heterogeneity this lineage within the OB is still unclear. A general analysis of the GFAP and S100β expression throughout the brain showed a high density of astrocytes in the OB (Emsley and MacKlis, 2006) and thus, considering OB astrocytes in functional studies is important to understand the specific role of glial cells in the SVZ-RMS-OB pathway (García-Marqués et al., 2010). Moreover, our data show that after postnatal or embryonic electroporation of the dorso-lateral part of the lateral ventricles there was no labeled glial cells within the OB. However, we could trace glial lineages in the OB, such as astrocytes, oligodendrocytes, and Ng2 cells after performing electroporations at E13 directed to the most rostral part of the lateral ventricles. Thus, these glial cells would appear to come from progenitors located within the OB. In addition, the distribution of glial cells within the OB in just one lateral region suggests that they may be generated following the radial glial processes, as in the cortex (García-Marqués and López-Mascaraque, 2013).

In summary, a better understanding of the neural cells generated in adulthood and the factors that control their proliferation, migration, and integration into neural circuits is crucial. Moreover, it is also essential to use clonal analysis so that single progenitor cells can be labeled and their progeny tracked in order to match progenitor cells to specific neural populations in the adult OB, including glial lineages.

## Author contributions

LM, Conceived and designed the experiments, writing the paper and obtained funding. MF, help in the design of the study, performed the experiments and writing the paper.

### Conflict of interest statement

The authors declare that the research was conducted in the absence of any commercial or financial relationships that could be construed as a potential conflict of interest.
