# Supplementary material for: Adult Olfactory Bulb Interneuron Phenotypes Identified by Targeting Embryonic and Postnatal Neural Progenitors
Source: Front Neurosci. 2016 May 9;10:194. doi: 10.3389/fnins.2016.00194 (PMC4860398; doi:10.3389/fnins.2016.00194)
Supplement: Supplementary file 1 [file Image1.PDF]

Supplementary Figures

Adult olfactory bulb interneuron phenotypes identified by targeting embryonic and postnatal neural progenitors

Maria Figueres-Oñate & Laura López-Mascaraque*

*Correspondence: mascaraque@cajal.csic.es

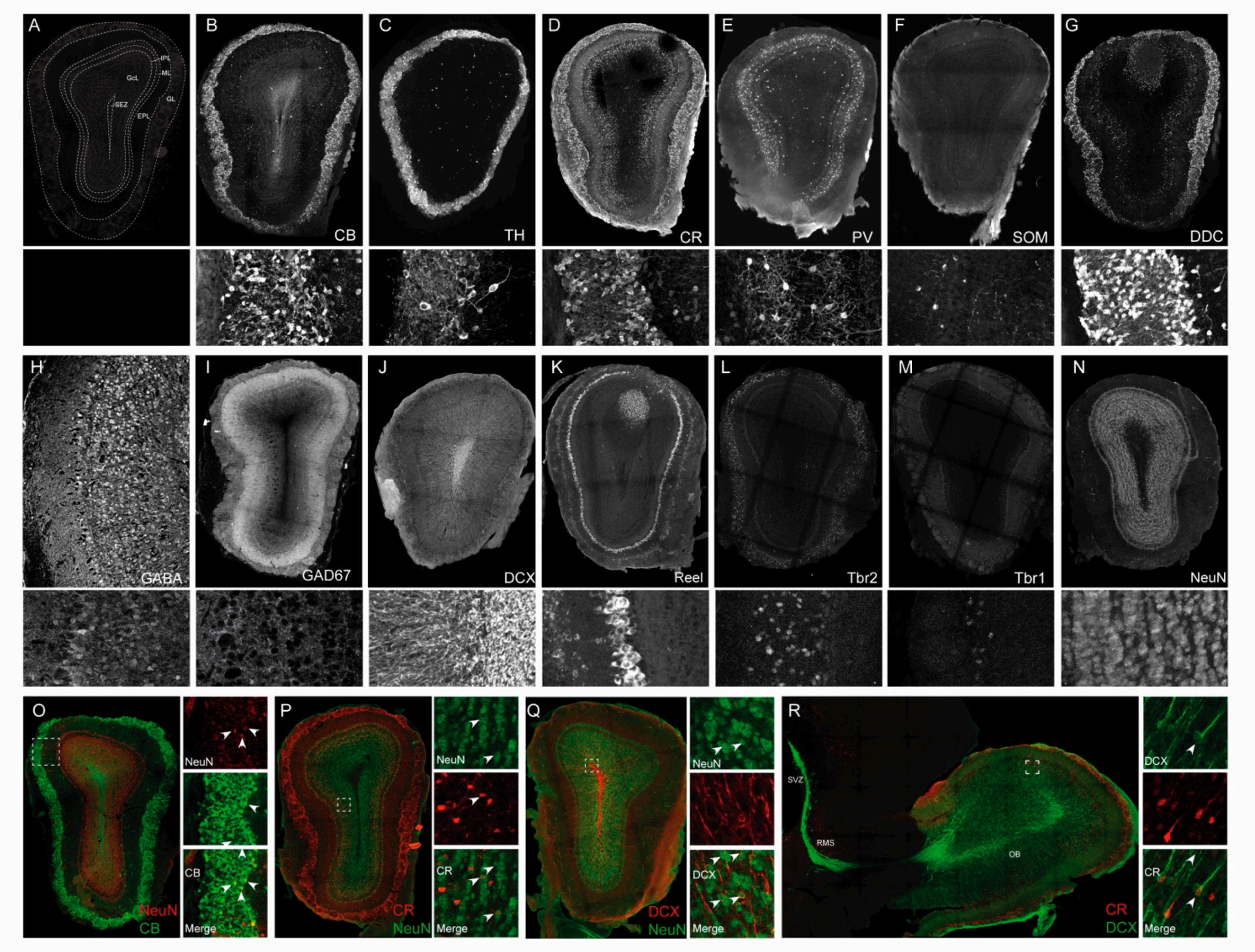

Supplementary Figure S1. Expression of different neuronal markers in the olfactory bulb. (A) Coronal section of the OB indicating the different layers. From inside-out, subependymal zone (SEZ), granular cell layer (GCL), internal plexiform layer (IPL), mitral cell layer (ML), external plexiform layer (EPL) and glomerular layer (GL). (B-N) Expression of the following protein markers within the OB: calbindin (CB, B), tyrosine hydroxylase (TH, C), calretinin (CR, D), parvalbumin (PV, E), somatostatin (SOM, F), dopa decarboxylase (DDC, G), GABA (H), GAD67 (I), doublecortin (DCX, J), reelin (Reel, K), Tbr2 (L), Tbr1 (M), neuronal nuclei (NeuN, N). (O-R) Dual immunohistochemistry for NeuN and CB (O), NeuN and CR (P), NeuN and DCX (Q) and CR-DCX (R). Arrowheads point to cells co-expressing both markers and asterisks to single labeled cells: SVZ, Subventricular Zone; RMS, Rostral migratory stream; OB, Olfactory bulb.

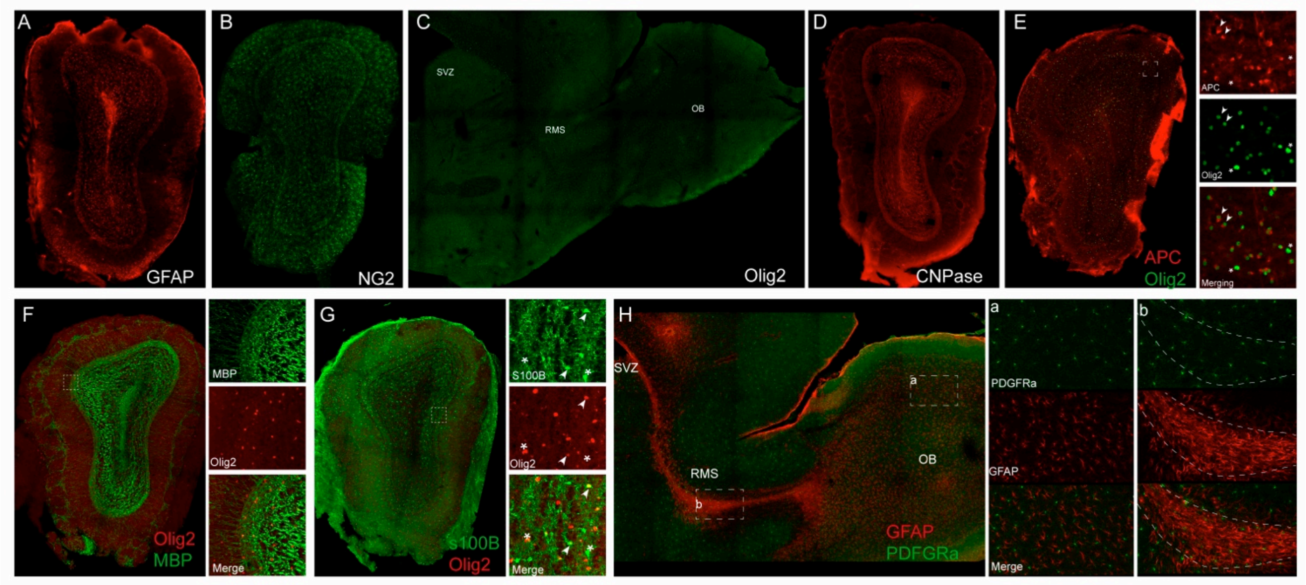

Supplementary Figure S2. Distribution of glial cells in the olfactory bulb. (A) Glial fibrillary acidic protein (GFAP) for astrocytes. (B) NG2-cells or polydendrocytes labeled with the Neuron-glial antigen 2 (NG2). (C) Olig2, the oligodendrocyte transcription factor 2 labels cells of the oligodendroglial lineage. Mature oligodendrocytes targeted either with CNPase (D) or co-labeled with APC-Olig2 (E, arrowheads). Olig2 targeted some cells from the oligodendroglial lineage that were not positive for APC (arrowheads, E). Co-localization of Olig2 with other mature oligodendrocyte markers, like MPB, was not clear (F). S100β and Olig2 co-localized in some cells (arrowheads, G) while the vast majority were from different cell populations (asterisks, G). GFAP and PDGFRα labeled different populations without overlapping (H). PDGFRα was homogeneously distributed in the SVZ-RMS-OB pathway, while GFAP was expressed strongly within the RMS and SEZ (H, a-b): SVZ, Subventricular Zone; RMS, Rostral migratory stream; OB, Olfactory bulb.

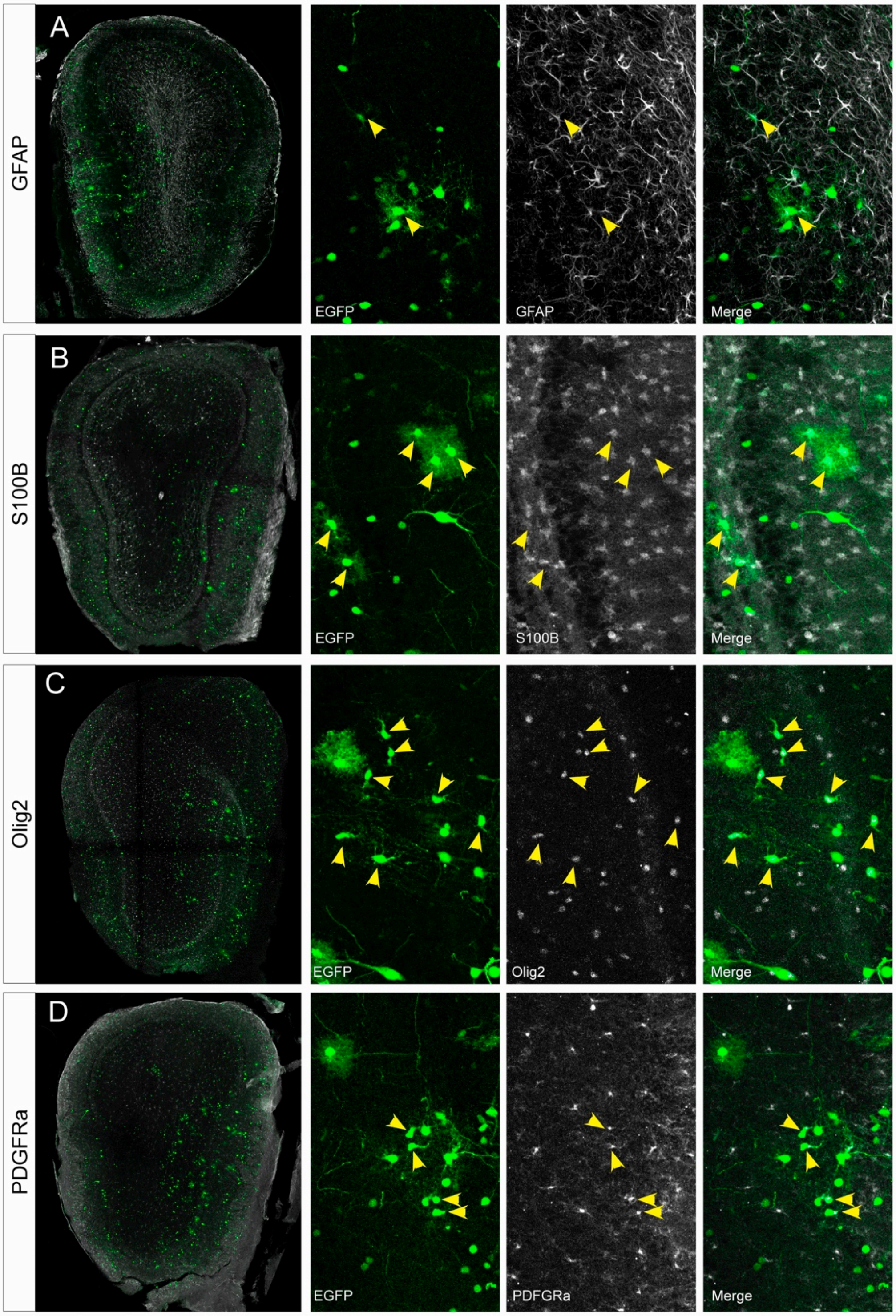

Supplementary Figure S3: Immunolabeling of different glial markers after IUE (E13) directed to the rostral ventricular surface. After electroporation of the most rostral part of the lateral ventricles at E13, glial cells were labeled in the OB 30 dpe. Arrowheads showed eGFP positive cells stained with the astrocyte marker GFAP (A). The astrocyte lineage was also assessed by staining with S100β (arrowheads, B). Oligodendroglial cells were also targeted by electroporation as witnessed with Olig2 (arrowheads, C) and PDGFRα (arrowheads, D).

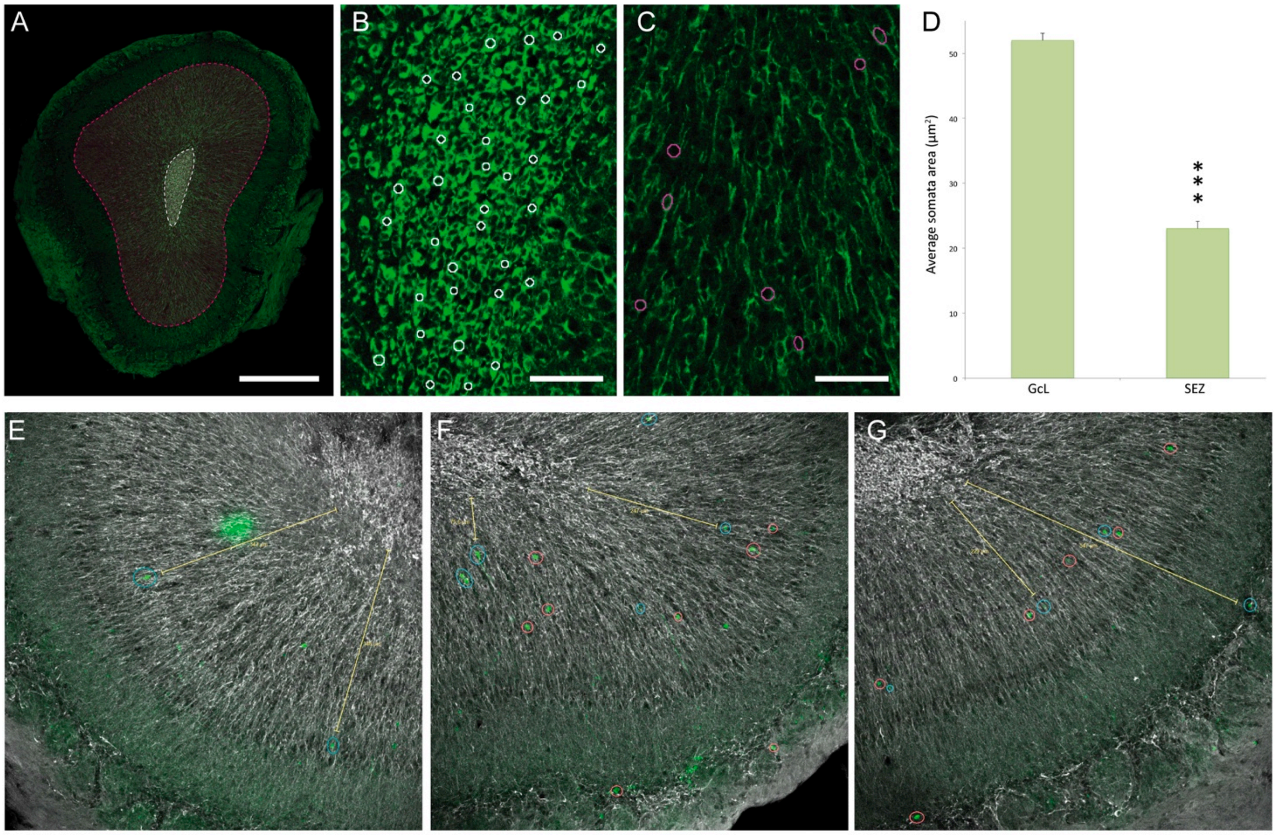

Supplementary Figure S4: Doublecortin immunolabeling. A representative coronal section of a P30 OB was selected to analyze the somata area of DCX positive cells (A). The area of the labeled cells was measured within the GCL (pink area in A) and the SEZ (white area in A). Somata of DCX positive cells were selected and counted in the SEZ (as shown in B, n=46) and GCL (C, n=60). Significant differences were seen when comparing the average somata size of labeled cells from the GCL (51.97±1.12 μm^2) and SEZ (23.02 ± 0.89 μm^2) (D). After electroporation at E15, some eGFP positive cells were also positive for DCX (blue circles in E-G) and the distance from the SEZ was determined in microns. Double stained cells were located in either the outer GCL (343-348 µm from the SEZ, E), close to the SEZ (75.6 µm from the SEZ, F) or surrounding the glomeruli (547 µm from the SEZ, G). Electroporated cells negative for DCX immunolabeling are indicated by a red circle. Scale bar 500 µm in A, and 50 µm in B,C.
